# Social Media: A Review and Tutorial of Applications in Medicine and Health Care

**DOI:** 10.2196/jmir.2912

**Published:** 2014-02-11

**Authors:** Francisco Jose Grajales III, Samuel Sheps, Kendall Ho, Helen Novak-Lauscher, Gunther Eysenbach

**Affiliations:** ^1^eHealth Strategy OfficeFaculty of MedicineUniversity of British ColumbiaVancouver, BCCanada; ^2^School of Population and Public HealthFaculty of MedicineUniversity of British ColumbiaVancouver, BCCanada; ^3^eHealth Strategy OfficeFaculty of MedicineUniversity of British ColumbiaPort Moody, BCCanada; ^4^Centre for Global eHealth InnovationTechna Institute and Institute Health Policy, Management and EvaluationUniversity Health Network and University of TorontoToronto, ONCanada

**Keywords:** social media, blogging, social network

## Abstract

**Background:**

Social media are dynamic and interactive computer-mediated communication tools that have high penetration rates in the general population in high-income and middle-income countries. However, in medicine and health care, a large number of stakeholders (eg, clinicians, administrators, professional colleges, academic institutions, ministries of health, among others) are unaware of social media’s relevance, potential applications in their day-to-day activities, as well as the inherent risks and how these may be attenuated and mitigated.

**Objective:**

We conducted a narrative review with the aim to present case studies that illustrate how, where, and why social media are being used in the medical and health care sectors.

**Methods:**

Using a critical-interpretivist framework, we used qualitative methods to synthesize the impact and illustrate, explain, and provide contextual knowledge of the applications and potential implementations of social media in medicine and health care. Both traditional (eg, peer-reviewed) and nontraditional (eg, policies, case studies, and social media content) sources were used, in addition to an environmental scan (using Google and Bing Web searches) of resources.

**Results:**

We reviewed, evaluated, and synthesized 76 articles, 44 websites, and 11 policies/reports. Results and case studies are presented according to 10 different categories of social media: (1) blogs (eg, WordPress), (2) microblogs (eg, Twitter), (3) social networking sites (eg, Facebook), (4) professional networking sites (eg, LinkedIn, Sermo), (5) thematic networking sites (eg, 23andMe), (6) wikis (eg, Wikipedia), (7) mashups (eg, HealthMap), (8) collaborative filtering sites (eg, Digg), (9) media sharing sites (eg, YouTube, Slideshare), and others (eg, SecondLife). Four recommendations are provided and explained for stakeholders wishing to engage with social media while attenuating risk: (1) maintain professionalism at all times, (2) be authentic, have fun, and do not be afraid, (3) ask for help, and (4) focus, grab attention, and engage.

**Conclusions:**

The role of social media in the medical and health care sectors is far reaching, and many questions in terms of governance, ethics, professionalism, privacy, confidentiality, and information quality remain unanswered. By following the guidelines presented, professionals have a starting point to engage with social media in a safe and ethical manner. Future research will be required to understand the synergies between social media and evidence-based practice, as well as develop institutional policies that benefit patients, clinicians, public health practitioners, and industry alike.

## Introduction

### Background

Social media are Web-based tools that are used for computer-mediated communication. In health care, they have been used to maintain or improve peer-to-peer and clinician-to-patient communication, promote institutional branding, and improve the speed of interaction between and across different health care stakeholders. Examples of social media applications in health include (but are not limited to) access to educational resources by clinicians and patients [1-3], generation of content rich reference resources (eg, Wikipedia) [4], evaluation and reporting of real-time flu trends [5], catalyzing outreach during (public) health campaigns [6,7], and recruitment of patients to online studies and in clinical trials [8-11].

A number of indicators suggest that the evidence for using social media in the health care context is growing; for example, the number of articles indexed on PubMed has nearly doubled each year for the last 4 years [12], social media policies are being adopted [13] and tested in various health care settings [14], journals are discussing how social media facilitate knowledge-sharing and collaboration [15,16], and theories on the social changes resulting from their adoption are being developed [17]. However, despite these useful insights, our collective understanding of how social media can be used in medical and health care remains fragmented.

### Objective

The aim of this narrative review was to gain a better understanding of how social media are being used in health care. Using a qualitative approach, this article uses case studies to illustrate where, how, and why social media are being used. The intent of this review is to allow different health care stakeholders the opportunity to make informed decisions on how to use social media and similar electronic-mediated communication tools as part of their daily activities.

## Methods

### Qualitative Method

Although literature reviews in medicine have traditionally followed positivistic epistemologies, we drew upon a different approach, the critical-interpretivist theory [18], to conduct this review. Our intent, more specifically, was to elucidate impact while illustrating, explaining, and providing the contextual knowledge of why social media are being used in medicine and health care. However, we did not intend to measure, quantify, or generalize results, as is the case with Cochrane Reviews. Ultimately, the knowledge synthesized herein will allow readers to decide for themselves where, how, and why they may use and implement these computer-mediated communication tools as part of their day-to-day activities.

### Data Sources and Knowledge Synthesis

This review used a number of traditional and nontraditional reference sources. It is not exhaustive due to inherent limitations that occur when trying to assess the medical and health-related grey literature situated within social media itself. Medline was searched using the search string in Textbox 1. Additionally, data from the Cochrane list of Web 2.0 resources [19], the Health Librarianship Canada (HLCanada) wiki [20], the Pan-American Health Organization’s Equity and Human Development Listserv, the 2008-2011 proceedings of the Medicine 2.0 World Congress on Social Media and Web 2.0 in Health, Medicine and Biomedical Research [21], and award winning blogs (eg, ScienceRoll) were used to supplement peer-reviewed resources. Where necessary, results were further supplemented with an environmental scan of the Google and Bing search engines.

Results were categorized based on social media service type (see definitions below) and, where necessary, further subgrouped as appropriate. The search above was conducted on January 1, 2012; however, all systematic reviews published after this date and up to July 2013 that fit these keywords were added to the literature.

**MEDLINE search string (modified from [12]).**

("second life" AND (virtual OR 3d OR immersive)) OR "virtual worlds" OR "web 3.0" OR "medicine 2.0" OR "health 2.0" OR "web 2.0" OR mashup OR "social media" OR Blog OR digg OR "del.icio.us" OR "social bookmarking" OR wikis OR folksonomy OR wikipedia OR flickr OR twitter OR youtube OR facebook OR myspace OR Linkedin OR FourSquare

### Definitions

Table 1 [22-26] presents a series of definitions and examples of different social media services.

**Table 1:** Categorical definitions of social media.

Service type	Definition	Example
Blog	Short for “web log”: a blog is an easy-to-publish website where bloggers (authors of blogs) post information and essays in sequential order [22].	WordPress, Blogger
Microblog	A tiny blog service that allows networks of users to send short updates to each other in less than 140 characters. Microblogs are considered a platform for information dissemination, social networking, and real-time communication [22].	Twitter, Identi
Social networking site	A social networking site is an online service, platform, or site that focuses on building and visualizing social networks or social relations among people, who, for example, share interests and/or activities. A social network service essentially consists of a representation of each user (often a profile), their social links, and a variety of additional services [23].	Facebook, MySpace
Professional networking site	A professional networking site is a type of social network service that is focused solely on interactions and relationships related to business or a person’s professional career [24].	LinkedIn, Sermo, Asklepios, Ozmosis, Drs Hangout, Doc2Doc
Thematic networking sites	Social networking sites centered on a particular theme; for example disaster response, nursing, etc. These share many aspects of, and operate as a community of practice.	Telehelp, Innocentive, 23andMe, PatientsLikeMeCureTogether
Wiki	Wikis are used to denote communal websites where content can be quickly and easily edited. Wikis support collaboration and information sharing; feature multimedia, such as video, slides, photographs; and allow anyone to edit or are password protected [22].	Wikipedia, Fluwiki
Mashups	A website that combines data and functionality from two or more services to create a new, value-added, service [25].	HealthMap, Google FluTrends
Collaborative filtering sites	A website where information is filtered or collected according to patterns. Techniques involving collaboration among multiple agents, viewpoints, and data sources are often used. These agents engage through a variety of sites, through a process called crowdsourcing, where the crowds join forces for a common purpose [26].	Digg, Delicious
Media sharing sites	A hosting service that allows individuals to upload and create galleries of photos, videos, and other digital media (eg, slide presentations). The host will then store them on a server and make them either publicly or privately available.	SlideShare, YouTube, Flickr
Other	Multi-User Virtual Environments, also known as Virtual Worlds	Second Life

## Results

### Summary

A total of 76 articles, 44 websites, and 11 policies/reports were reviewed and synthesized. Ethics, professionalism, privacy, and confidentiality, as well as information quality were recurrent themes throughout the literature. These are synthesized throughout the manuscript and emphasized in the discussion section. Prospectively, our results are presented based on the definitions of social media categories presented in Table 1.

### Blogs

The first (Web 2.0) social media were developed in the late 1990s in the form of Web-logs (a term which was later shortened to blog). Web-based software platforms like Open Diary enhanced accessibility of content by allowing any existing or new users in the lay public to create a communal website where opinions about any topic could be voiced to create communal, collaborative dialogues. Blogs foster open access to information (both opinions and facts), contribute largely to the number of new websites created on the Internet and are often picked up by mainstream media, which makes them an important vehicle for social change [27]. For example, Paul Levy, the former President and CEO of Beth Israel Deaconess Centre in Boston, MA, was one of the early adopters among health care executives who, as a public authority with significant power, wrote a blog to make his reflections and decisions at the hospital transparent to all [28].

Although the literature on the use of blogs in Medline is growing, only one study that formally evaluated the attitudes, perceptions, and realities of the medical blogosphere was identified. Kovic and colleagues [27] conducted an online survey of medical bloggers and found that successful medical bloggers are most often highly educated writers (with masters or doctoral-level degrees) who are faithful to their sources and readers and are motivated to influence how others think by sharing their practical knowledge or skills in a creative manner.

Educational institutions in health care (eg, The Mayo Clinic) have used blogs to foster reflective peer-to-peer learning, which allow for open discussions and a formal log of medical training, as well as the implementation of new protocols [29]. Many hospitals also use blogs for branding and community outreach to (1) advertise their facilities [28] (such as featuring the newest device or test that they competitors do not possess), (2) share positive patient experiences [30] (such as a Mayo Clinic’s Piano Foyer Video where 2 unrelated patients meet and play the piano together while in hospital), or (3) feature well-known physicians who treat famous people or athletes [31]. Hospitals also use blogs to disseminate disease-specific information to supplement leaflets or handouts for patient education [32].

Blogs have been used in clinical research for clinical trial recruitment and data collection, allowing patients to ask questions about the trial procedures, risks, and incentives while maintaining an anonymous, non-threatening environment [33]. Mayo Clinic has also used blogs focused on major depressive disorder to request feedback on the patient experience and some of the complementary medicine practices they follow [1].

Patients have also been using blogs creatively to monitor and share their own patient journeys. For example, SixUntilMe (named after the age at which the author, Kerri Morrone Sparling, was diagnosed with diabetes) features the life of a patient living with type 1 diabetes, discussing topics like insulin pumps, continuous glucose monitors, and diabetes advocacy [34]. Cancer patients have also used blogs to share their experiences with chemotherapy. Dave deBronkart, a well-known e-patient advocate, used his blog to inform family members and his attending (family) physician of changes in tumor growth from a self-created spreadsheet of radiology reports of tumor size data [35]. Also, in May 2011, the Vancouverite Derek K Miller had a friend post his auto-obituary after dying from stage 4 metastatic colorectal cancer. His self-obituary blog post [36] “went viral”, receiving more than 4 million views in the 4 days after his death—a rather startling example of the potential reach of health-related social media.

**Figure 1:**
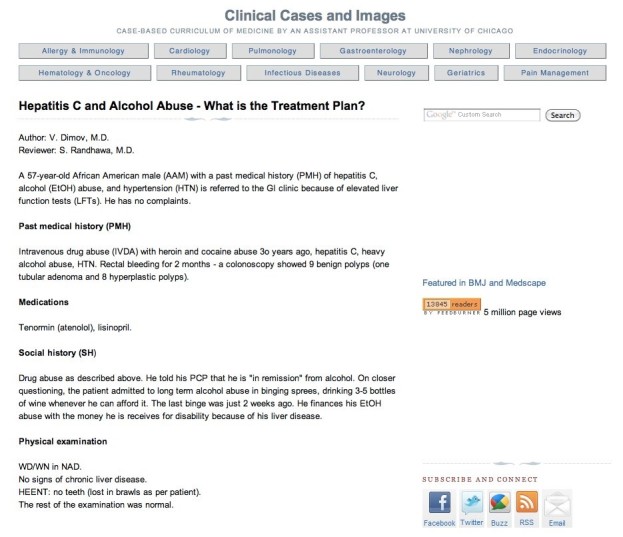
A sample rounding blogging case.

Blogs have been used by health care workers for peer-to-peer communication and knowledge exchange such as virtual rounds. The Clinical Cases Blog [37] is prototypical of the medical blogosphere, as it features cases in allergy and immunology, cardiology, pulmonology, gastroenterology, nephrology, endocrinology, hematology, rheumatology, infectious diseases, neurology, geriatrics, and pain management. Moreover, this blog also has a special section on admission note templates (eg, congestive heart failure), procedure guides, and related material. Figure 1 displays an example case from this blog.

With regard to disease and epidemic outbreak tracking, citizen-report photo blogs have been used to inform hospitals of incoming mass casualty events (eg, Hudson River plane landing) [38]. Equally interesting, is how the US military has used natural language processing (where computers evaluate meaning) to automatically filter and retrieve information on blog posts by military servicemen as a means to monitor emotions and posttraumatic stress disorder after operational deployment [39].

There are many other examples of medical blogs in addition to the ones discussed. MedGadget [40] showcased a range of interests in 2010 with their annual (winter) medical blog competition using a public voting system. To see the winners, further illustrating how blogs are used in health care, see Multimedia Appendix 1.

The Really Simple Syndication (RSS) Web standard has facilitated the broad adoption and dissemination of blogs. RSS allows software, known as RSS readers, to pull content and create an email-like inbox of blogs and other websites (eg, PubMed) that are frequently updated. This is useful when a user wants to create a customized “feed” of information that is relevant to their interests and classify it accordingly, for easy retrieval in the future. Among the most notable RSS readers are Apple Mail, iGoogle, and Bloglines.

Overall, blogs are the oldest, most established, and evaluated form of social media, with articles as early as 2004 noting their use in medicine and family practice [41]. A number of peer-review articles on blogs have also been published. These mainly note their effectiveness how they can be used to disseminate best practices [42], their applications in assessing clinical knowledge learned [43], and how they can be used to promote reflection and professional development [29].

### Microblogs

The most dynamic and concise form of information exchange on social media occurs on microblogs. These short 21^st^century telegrams allow users to view a large number of updates, of brief content, over a short period of time. Today, a large number of microblogging platforms exist, catering to audiences varying from the corporate world to teenagers; however, Twitter is and has remained the most prominent service on the market. Twitter updates are known as tweets.

Newcomers to Twitter often perceive the character restriction as a barrier to communication; however, this misconception usually decreases with repeated use, as tweets are easily supplemented with shortened hyperlinks to other digital media, such as videos or websites. Historically, a 140-character limit was chosen to allow interoperability with SMS (short message service) text messages.

“Tweeps” (people who tweet) also often use other services that connect to the Twitter platform (eg, TweetDeck, HootSuite), which allow them to organize their tweets, manage information, and see website previews or pictures without having to click on a link and open a new Web browser window. Some of these services also sort, filter, and curate tweets, allowing a user to see updates related to a particular topic (eg, health care issues). In turn, this has caused a new technical tweeting language to emerge. A summary glossary of this language can be found in Multimedia Appendix 2, adapted from the Twitter Glossary.

Tweets and tweeting styles can be classified in three broad categories [44]. Substantive tweets are independently understandable (eg, a tweet with an abridged title or author of a paper, a brief comment, and a link to the publication, or a headline teaser to a blog). Conversational tweets are fragments of a new or ongoing conversation that draw on professional or personal interests or comment on current events. Finally, there are hybrid tweets, which are substantive and conversational at the same time (eg, “discussing my supervisor’s newest Nature publication at the Mahoney and Sons pub”).

In medicine and health care, there have been over 140 reported uses for Twitter [45]. There have been some interesting applications of Twitter in medical education. The Pennsylvania State College of Medicine has used Twitter to augment peer-to-peer and instructor-to-student learning [46] by stimulating topic discussions, providing feedback on critical thinking, conducting course evaluations, disseminating writing prompts, soliciting class responses, and monitoring student progress. Second, a junior doctor and a medical student started a Twitter Journal Club [47] that functions in the same manner as traditional journal clubs, except that the means for discussion is Twitter. By using a combination of blog posts, where the paper and discussion questions are posted in advance, along with the hashtag #TwitJC, students, doctors, and anyone interested in the subject can engage and interact in a meaningful way. The club meets every second Sunday evening.

The use of Twitter at conferences can also be seen as a medical education application [48]. In this case, Twitter functions as a tool to discuss and enhance the speaker’s presentation in real-time through the comments of the audience. The Medicine 2.0 conference series has pioneered the use of Twitter-screens, displaying the tweets of the audience alongside the presenter slides.

In terms of health service delivery, 2 physicians have used @tweetspreekuur since October 2009 for primary care consultations [49]. Using the concept of learning by doing, the service was launched with little planning. After 1 year, their tweeting practice has shown that consultations encompass all areas of primary care, though the main reasons for contact are advice, reassurance, and triage. Typically, Tweet exchanges vary from one to eight tweets in length and about one third of the communication takes place publicly, while the other two-thirds takes place through direct messages (which are not public). Pictures of skin and genital-related problems have also been sent to the service. Presently, @tweetspreekuur is run on a voluntary basis (there is no reimbursement to the physicians who run the service) and the “attending physicians” involved stipulate that their success is due to language (consultations take place in Dutch, limiting their audience) and the option for patients to continue the consultation through a secure online platform, only available in the Netherlands. Today, preliminary research [50] suggests that Twitter has been effective at providing access to care at a low cost, that running the service is fun and entertaining for the providers, and that the level of user satisfaction is high.

In this section, it is important to draw attention to hashtags, which are a form of information curation that allow people to find tweets related to a particular discussion or topic. Among the most common are #HCSM or Health Care Social Media and its Canadian (#hcsmca), European (#HCSMEU), and Latin American (#HCSMLA) variants, #Med2, #MDChat, and #Health20. For example, a tweet that has both the #Med2 and #HCSMCA hashtags will be read by people who filter tweets because of their interest in information related to social media in Canada, as well as the Medicine 2.0 conference series.

On the negative side, there are a large number of bots (short for “robots”) that re-tweet and spam Twitter users who use particular words, phrases, or hashtags in order to increase their user reach and digital footprint. Fortunately, these phony users are typically removed by the Twitter service relatively quickly due to the “report spam” feature on the site. Also, due to the limited size of a person’s profile, it is essential that Twitter users double check the identity of the person whom they are communicating with, as it is easy to create a fake profile and communicate with an unknown charlatan on the service.

### Social Networking Sites

Although different types of social media are often categorized as social networking sites, for the purposes of this paper, social networking sites are defined as Web-browser and smartphone accessible services that allow users to create social connections in a public or semi-public form (through the use of profiles) in order to share information updates with other site users. Wikipedia, the online user-generated encyclopedia, further expands on this definition with a number of concepts, as can be seen below [23]:

A social networking service is an online service, platform, or site that focuses on facilitating the building of social networks or social relations among people who, for example, share interests, activities, backgrounds, or real-life connections. A social network service consists of a representation of each user (often a profile), his/her social links, and a variety of additional services. Most social network services are Web-based and provide means for users to interact over the Internet [through] instant messaging. Online community services are sometimes considered as a social network service, though in a broader sense, social network service usually means an individual-centered service whereas online community services are group-centered. Social networking sites allow users to share ideas, activities, events, and interests within their individual networks.

The majority of the peer-reviewed literature on social networking is centered on issues of maintaining professionalism, ethical practices, identity, and privacy. However, given that these subjects apply to all types of social media, they are addressed in the discussion section. Cyberbullying is also a common topic of discussion of the literature in this space; however, it is out of scope for this paper.

An iconic paper by Farmer and colleagues (2009) [51] evaluated the relationship between Facebook groups and common medical conditions. They found that the most common type of groups on Facebook were centered on specific medical conditions (eg, malignant tumors), peer-to-peer support, and fundraising for support groups, organizations, and individuals. Farmer and colleagues also found that researchers used Facebook to aggregate themselves into a “network” for dissemination of their research to other researchers and health care providers. They also identified the existence of self-aggregated negative-behavior support groups, mainly centered on the promotion of excessive alcohol consumption.

Similarly, Bender and colleagues (2011) [52] found that the majority of those who use social networking sites use them to form self-aggregated interest groups. Within a single disease, breast cancer, a search on Facebook revealed over 600 support groups organized around four central themes: fundraising, awareness, marketing, and general support. General support groups were not used as an adjunct to supportive care nor did they serve as a general form of patient-to-patient support; rather, they were most often created by a user (or family member) with cancer as a means to keep friends and family members updated on their treatment and, at the same time, receive supportive feedback. Bender et al [52] also noted that their results may be skewed because they were able to analyze public groups only, which had very few user contributions as a whole. Furthermore, the technical architecture of Facebook, which makes it difficult to have a fictitious profile when compared to other (more open) social media such as Twitter, may also be responsible for skewing the data. This is a general limitation of research on social media sites—all closed profiles and private conversations cannot be evaluated unless the actual patient discloses the content of these interactions, thus this literature review did not find any formal research comparing “closed” groups on Facebook.

Another interesting use of Facebook groups occurred in Taiwan [53], where a well-known emergency physician blogger created a public group to ask his colleagues as to how they could improve patient wait times in the emergency room. In less than a month, the group grew virally, with the majority of emergency department staff from around Taiwan proposing solutions. Eventually, the group received so much attention that the Minister of Health himself (and his staff) joined the group and commented directly, using the comments from its 1500 plus group members to make policy decisions. This culminated with the minister making visits to emergency departments in ten different cities and promising to initiate a dialogue to improve funding and reduce wait times in emergency departments in collaboration with the Taiwanese Bureau of National Health Insurance.

An important facet of most social networking sites is that third-party applications (apps) can be created within these services. Third-party applications work through the integration of application programming interfaces (APIs) that allow outside software and data to be visualized. In Facebook, the most prominent of these is Zynga’s Farmville Game, which allows people to create a virtual farm and, by interacting with other Facebook friends, acquire a virtual currency that can be used to buy virtual goods, such as tractors or animals.

**Figure 2:**
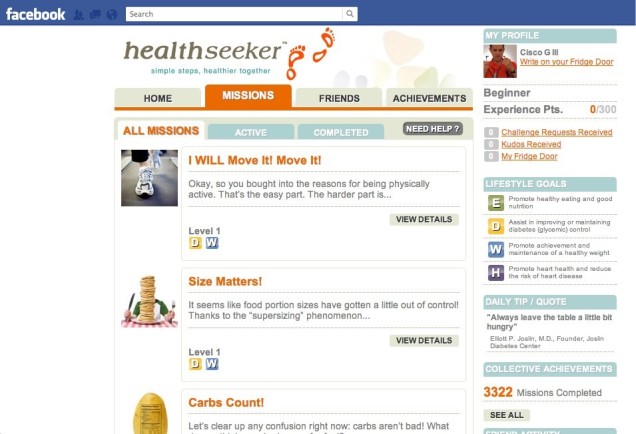
The HealthSeeker Diabetes Education App on Facebook.

Similar examples within health can be found in an article by Fernandez-Luque and colleagues [54] that searched for and evaluated these “apps” within Facebook. This research found that less than 30% of listed applications were real and the remaining 70% were non-functioning “spam”. In their evaluation of the 56 working applications, Fernandez-Luque and colleagues found that these software were thematically centered on fitness and weight loss, specific health conditions (eg, diabetes) education, smoking cessation, and fundraising for health and research-related activities. The most notable included “Get up and move”, which allows people to challenge their friends to engage in physical activity and report on it after they have completed it; the American Heart Association’s “START” application, which was part of a heart portal and allowed users to answer questionnaires on cardiovascular health and upload the data to a health portal; and HealthSeeker, a diabetes management education app (see Figure 2) allowing users to learn how to better manage their diabetes and gain “points” that could be used for incentive draws in the process. Although not specifically named, two other applications were also described that allowed users to make appointments for blood donations. Only one application was made for physicians, which was used as a forum to answer patient questions.

Within the research realm, Bull and colleagues published a reflective case study that discusses the ethical questions that emerged during a Facebook-based randomized controlled trial of preventative HIV education for high-risk teens in the United States [55]. They found that maintaining ethical principles was the most difficult part of using Facebook for research. In particular, maintaining beneficence, improving knowledge and information comprehension, ensuring equity of special populations, and safeguarding confidentiality and security were the largest challenges to the study’s implementation. To overcome these problems, Bull and colleagues referred study participants from a Facebook fan page to an external website, which was congruent with the US Health Insurance Portability and Accountability Act (HIPAA) and their Institutional Review Board’s requirements. Bull and colleagues concluded by recommending that researchers who plan to collect data from social networking sites consider whether the social networking service is the appropriate vehicle for participant recruitment, that they offer multiple venues for participants to provide informed consent, and that all data are safeguarded behind secure firewalls, preferably outside the original social networking site.

### Professional and Thematic Networking Sites

**Figure 3:**
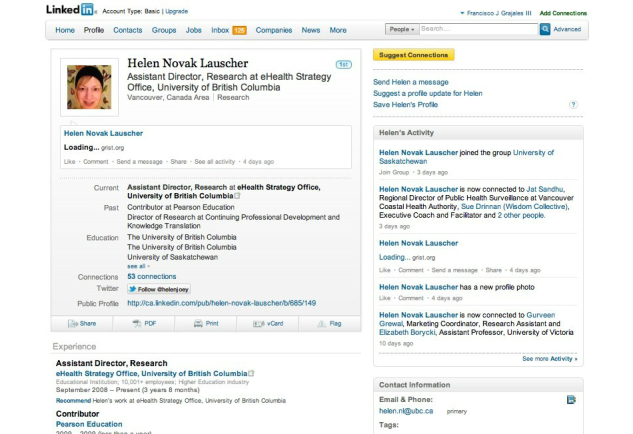
A public profile on LinkedIn.

Professional networking sites are aimed solely for interactions related to a person’s professional career or business. LinkedIn is the most popular of such sites and does not solely focus on medicine or health care; it allows people to publicly display a curriculum vita along with personal and institutional affiliations. Unlike Facebook, which allows people to “friend” each other, LinkedIn uses connections, which publicly show people that have worked together or know each other. Should a person be new to the site, connections can also be used to visualize the number of degrees of separation between 2 or more people. Figure 3 displays a public profile on LinkedIn.

A number of health care specific professional networking sites also exist, including Sermo, Asklepios, Doctors’ Hangout, Ozmosis, Doc2Doc, and others, which try to recreate the intimacy of the “physicians’ lounge” in an online environment. These sites most often require the clinician to submit their credentials to a site gatekeeper, thus creating the perception of an elitist forum that is “safe” from patient’s eyes. Discussions in these sites typically range from dating in a medical environment, ethics, clinical trial and medication reviews, biostatistics, and specific treatment options. A combination of business models are also used by these sites, which vary from financial sponsorship by a professional association (eg, Asklepios by the Canadian Medical Association), advertisement, anonymized data vending to external stakeholders (eg, insurance companies, pharmaceutical companies, etc), commission on prizes offered by companies trying to solve a particular problem (eg, InnoCentive), and research by external stakeholders (eg, surveys on physician medication prescription habits).

Thematic networking sites are analogous to professional networking sites but centered on a particular theme. These include telemedicine (eg, Telehelp), informatics (eg, Health Informatics Forum), nursing (eg, SocialRN), genomics (eg, 23andMe), and patients (eg, PatientsLikeMe), among others. Of particular interest are patient thematic networking sites, as a number of these sites collect, aggregate, and visualize patient data to promote patient-driven research (research that was initiated by a patient and used to collaborate with other patients with the same or a related disease) [56].

**Figure 4:**
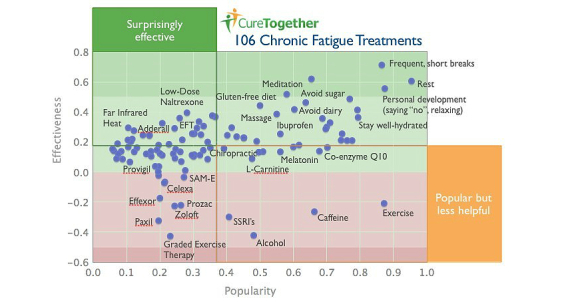
CureTogether’s page on chronic fatigue syndrome.

One site that promotes patient-driven research is CureTogether. It collects a number of health metrics including weight, caloric intake, sleep, exercise, and other disease-specific indicators [57]. Although the site is not meant to constitute medical advice, it allows patients to summarize statistics on treatment efficacy, side effects, and causes of disease, ultimately helping people make more informed treatment decisions. For example, on its chronic fatigue syndrome page, CureTogether has amalgamated responses from over 1300 patients, encompassing nearly 7000 data points on effective treatments. Indeed, it is this “crowdsourced” or collective wisdom that is believed to combat single stakeholder bias. This approach remains strenuously contested by physicians and the public alike, as it is difficult to prove, in terms of accuracy and validity, that third parties have not intervened in how results are displayed to users. Figure 4 displays a summary page on different treatment effectiveness for chronic fatigue syndrome.

### Wikis

Wikis are easy-to-publish websites that can be quickly and easily edited by multiple users; they feature both text and multimedia content. (The term wiki was borrowed from the Hawaiian language and it stands for quick; it is a metaphor of the speed with which information can be accessed, added, and edited on a wiki.) Within medicine, the most commonly cited and used wiki is Wikipedia, which receives over 150 million page views per month, with the top 200 medical articles receiving over 100,000 monthly page views [58]. Additionally, the Health Library Wiki of Canada (a University of British Columbia Library initiative) lists over 61 medical wikis and wikibooks available to health care professionals and patients alike.

Although Wikipedia’s accuracy and completeness are often debated, as is the content of many wikis, research by Clauson and colleagues [59] compared Wikipedia’s drug information with the Medscape Drug Reference. This research found that Wikipedia had very few factual errors and that it included approximately 76% of the content found in Medscape (a validated and trusted information source) [59]. Wikipedia was also found to foster quality/accuracy improvements over a period of 90 days, due to crowdsourcing, which was not the case with Medpedia, due to their more restrictive editorial process [59]. In their concluding remarks, Clauson et al noted that Wikipedia was a good starting point for health information consumers, despite being narrower in scope, less complete, and with some errors, mainly of omission, than Medscape.

In terms of its editorial membership, a recent article published by some of Wikipedia’s medical editorial team (a self- and peer-selected group of WikiProject contributors who work together to improve Wikipedia) [58] noted some of the dilemmas faced on this digital space. Among the weaknesses noted is that some people and organizations (eg, the pharmaceutical industry) have used the site to advance their personal and corporate mandates. Also, Wikipedia’s user architecture uses a self-selected pseudonym for authors and editors of content, which makes it very difficult to verify the expertise or credentials of the contributors. Among its strengths, Wikipedia has an effective rating system analogous to peer review. Identification of promoting an article to “Good Article Status” requires the independent review by at least 1 editor, and to “Featured Article Status”, review by a group of editors. Becoming an administrator on Wikipedia is also no easy task, as user rank promotion is subject to a public voting system where the date and number of articles edited and authored are evaluated by other Wikipedians. In addition, IP addresses of the votes are cross-checked to prevent a single person from self-nomination. Wikipedia has a style manual [58], which, for example, recommends that drug dosages be excluded from the site in order to prevent patient harm. There is also an elaborate process of content verifiability if disputes arise between editors to ensure successful conflict resolution and neutrality of the information.

Other notable wiki use for public health include the World Health Organization’s (WHO) International Classification of Diseases 11 update experiment [60], which opened the International Classification of Diseases system used around the world, allowing clinicians to consider and add new codes before the WHO ratified the new classification strata through its internal processes. Similarly, the Medskills wiki, which is a wikibook that compiles physical assessment techniques, allows students to learn them without having to buy expensive textbooks. Wikisurgery is a free surgical encyclopedia, and OpenWetWare features a number of laboratory procedures to facilitate the learning steps of complicated laboratory techniques (eg, a DNA polymerase chain reaction).

A large variety of wiki-like software exists on the Internet. These include Google Documents (GDocs), which is a word-processing program analogous in functionality to Microsoft Word but with the added benefit of simultaneous synchronous user collaboration and automatic document publishing as a webpage [61]. Similarly, Etherpad, is an open source, low bandwidth, massive user (>30) collaborative writing tool, particularly suitable for simultaneously working on a document during (tele)conferences and meetings with a large number of attendees, due to its minimalistic interface [62].

The use of wikis in health has significant challenges. These include attracting and maintaining a critical mass of content contributors and editors to maintain accuracy and currency of content, and dealing with wiki damage, which occurs through (human or robot) spam, link rot, deletion of information such as medication side effects (despite the ability to see a history of changes, similar to MS Word’s track changes feature), and the deliberate insertion of misinformation (eg, neutraceutical companies noting that their products can cure cancer and other similar claims [58]).

### Mashups

Mashups are combinations of two or more Web services that use APIs to create a new service or functionality. The term was borrowed from the music industry, where separate music tracks are combined by DJs to create a new song. The first medical mashups originated in the form of analog Geographical Information Systems, similar to John Snow’s map of the cholera outbreak in London in 1854 [63]. Today, a large number of medical mashups exist (see Table 2) ranging from infection disease visualization (eg, HealthMap) to PubMed search engines, which use semantic technologies to find peer-reviewed articles that closely match an author’s written block of text (eg, ETBlast3).

**Figure 5:**
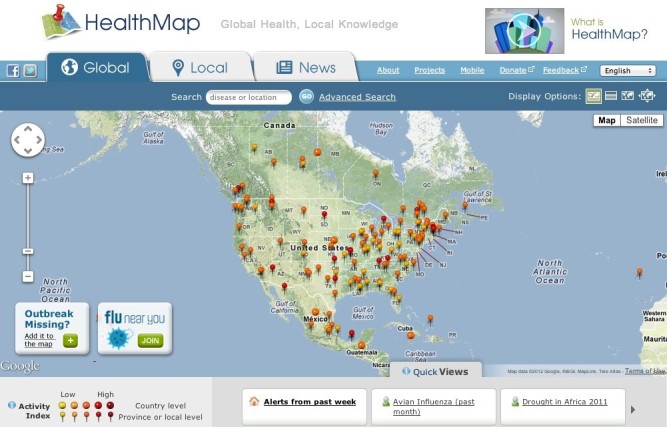
North American outbreaks in the HealthMap Mashup.

HealthMap is an example that illustrates both the importance and need for mashups by large organizations. Financed by the Google Foundation and supported by the Canadian Institutes for Health Research, US Centers for Disease Control, and the National Library of Medicine, HealthMap uses Fisher-Robinson Bayesian filtering to aggregate information from the WHO’s Information System (WHOSIS), the Program for Monitoring Emerging Diseases (ProMED-mail) databases, Geosentinel (the global surveillance program from of the International Society of Travel Medicine), the World Organization for Animal Health, the European Centre for Disease Prevention and Control, Baidu and Baidu News, and Google News [64]. HealthMap combines a very large variety of data sources and APIs (eg, Google Maps, Google Translate, etc) to create a highly powerful information resource that can be “zoomed” all the way to relatively small geographic regions (eg, suburbs). When looking for disease outbreaks, all details are dependent on the source data, which means that while some geographic regions may display a high level of information, others may not, which is a general weakness of the site. Figure 5 displays the HealthMap mashup for North American disease outbreaks.

**Table 2:** Some well-known health-related mashups.

Site name/Address	Brief description
Google Earth	Typically known as a world visualization website, Google Earth features time-enabled maps in order to track worldwide flu trends by using google.com symptom search queries.
Healthmap.org	HealthMap, led by a multidisciplinary team in Boston’s Children’s Hospital, uses informal data sources for real-time world-wide disease surveillance and outbreak monitoring.
Sickweather.com	Sickweather uses a patent-pending algorithm to aggregate data from Facebook and Twitter along with self-reported data in order to forecast, track, and map a number of illnesses around the world.
Whoissick.org	Whoissick aims to provide current and local sickness information to the public. Although it was one of the first disease visualization mashups, today the site has little data and is likely to be defunct in the near future. The main reason is a lack of a community, which provides data to the site. Whoissick also does not reveal which data sources it uses to visualize disease and symptom outbreaks.
etest.vbi.vt.edu/etblast3	eTBLAST is an article search engine that looks for peer reviewed articles, such as those on PubMed, which resemble any block of text. Thus, one can write a paragraph and look for articles, which will support the premises noted. This mashup is a project of the Innovation Laboratory at Virginia’s Bioinformatics Institute.

### Collaborative Filtering

Collaborative filtering sites are websites that allow multiple users to tag or classify and crowdsource information to create a user-based, bottom-up folksonomy (a user-generated, unstandardized taxonomy). Today, a collaborative filtering feature can be found in most blogs (eg, to classify blog posts into one or more subjects or themes), microblogs (eg, through the use of hashtags), wikis (eg, to find related articles), and media sharing sites (eg, to find similar pictures or videos). Content tags are used to facilitate information filtering and when combined with a semantic (text analysis-based) algorithm, which prevents typos from being incorporated into the folksonomy as a new classification term, are powerful data curating tools. Three prominent collaborative filtering sites are Delicious, Digg, and Connotea [20].

Digg is a social news site that allows registered users to give a “thumbs up or down” on a news story. By doing so, articles are pushed up or down on the site’s landing page, allowing readers access to the “best” content as voted by the community. Users also have a comment and follow feature, allowing them to access other user’s views and subject interests. Moreover, Digg also integrates a number of APIs, such as Facebook Connect, allowing users to share articles and their views on them to other social media sites. In health care, Digg can be particularly useful to policy makers and hospital administrators who wish to get information on the latest outbreaks and health-related news in their local community, as the site allows access to the “highest regarded sources” as voted by the Digg user community.

Delicious is a social bookmarking service that allows users to store, share, and discover Web bookmarks. Its primary allure is a user-chosen tagging system, which allows people quickly to filter through a large number of bookmarks in a short period of time. Also, due to its Web-based nature, users can access their bookmarks from any computer with Internet access. Other features include public and private bookmarks, groups, and similar and popular link suggestions, which allow for collaboration. In medicine and health care, Delicious can be used to create high quality collaborative knowledge repositories (eg, with resources from WHO, Centers for Disease Control [CDC], Health Canada, etc) that are centered on a particular topic (eg, a treatment) and can be easily accessed and by a select (or open) group of people (eg, a hospital department).

Connotea is a free online reference management site for clinicians and scientists. It allows users to share and organize their references and receive updates as to what colleagues are reading and adding in their reference libraries [65].

It should be noted that the Web traffic rating site, Alexa, shows that despite collaborative filtering sites’ usefulness, they are losing popularity and market-share in attracting new and maintaining old users due to the rising integration of a tagging feature in other social networking sites.

### Media Sharing Sites

Media sharing sites comprise a large palette of social media tools that are optimized for viewing, sharing, and embedding digital media on other Web services. They share a large number of attributes with other social media—profiles, friends, comments, and private messaging/sharing of content—but their success is determined by the type of content uploaded and shared. Views are often not necessarily related to the quality of the media or its accuracy, as viral content sharing may be erroneous or have poor resolution. Most often, a site’s catchy title [66], amusement level (eg, a baby panda sneezing and the mother panda being scared by the baby) [67], or relevance to current affairs is what affects its “virality” [68].

Media sharing sites, nevertheless, are great resources for knowledge translation (eg, the Ken Jong CPR video [69]), community building (eg, multiple sclerosis patient-to-patient videos [70]), marketing (eg, Viagra commercials and information pamphlets [71]), research (eg, video explaining patient rights [72]), education (eg, medical skill demonstration videos and summary sheets [73]), and branding (eg, Mayo clinic patient playing piano video [74]). They are also easy to use, have no cost (for non-premium accounts), and are accessible from both desktop and mobile devices. Table 3 illustrates the different types of media sharing sites, a common example, and their description.

A number of articles have been published on the use of media sharing sites, primarily focusing on the use of audio and video podcasts for health professional education, patient-to-patient communication, and public health campaigns.

Within the patient-to-patient communication realm, Fernandez-Luque and colleagues (2009) evaluated the comments from a random sample of YouTube videos created by patients with multiple sclerosis [75]. They found that virtual communities emerged through the “comment” feature of the site, with patients responding to each other’s videos, documenting the progression of their disease, and endorsing certain medications that should be used as a last resort for the treatment of a disease (eg, Tysabri). Of concern was the direct interaction of the pharmaceutical industry with patients, requesting them to contact pharmaceutical reps to become champions and public advocates for particular medications.

Similar research by Keelan and colleagues at the University of Toronto featured a characterization of available immunization information in YouTube [76]. They found that the most commonly discussed vaccine topic was childhood vaccines (accounting for 25% of the total vaccine videos) with the most specific vaccine topic being HPV (human papilloma virus). Overall, negative videos (eg, those that contradicted the Canadian Immunization Guide) were more likely to receive a higher number of views and user ratings, and accounted for approximately 50% of total YouTube immunization videos. (YouTube does not discriminate video ranking based on content unless the video violates copyright policy; in which case, it is removed. Generally, the number of times a video is viewed is the main driver behind search result rankings.)

Media sharing sites have also become encyclopedic resources. Among the most notable are the Khan Academy, which hosts over 3000 videos and practice exercises in everything from algebra to medicine and health care, and the Doctors’ Channel, which hosts online videos for a variety of health care professionals featuring content about continuing medical education, medical news, and health care–related entertainments.

Negative effects from media sharing sites have also been reported [75]. YouTube, copyright infringements are common; however, copyright owners can opt in to receive a share of advertisement revenue to keep content online. Also, few child protection initiatives have been implemented on these services. For example, if one types “proana” and “thinspiration” on YouTube, over 27,000 collective videos can be recalled. These can lead individuals to cause themselves harm by applying information on how to support anorexia and bulimia, as well as finding other equally ill individuals who become supporters in maintaining a disease-prone lifestyle [77].

Finally, it should be noted that not all content available on media sharing sites is available to anyone with an Internet connection—some countries block access to these sites (eg, China). Notwithstanding, it is also important to consider that the high prevalence of mobile phones has broken the capture and upload barrier for these sites, which means that if an organization or individual is not constantly monitoring their online presence, it is easy for an individual to take a video of themselves complaining about the care they have received and upload it onto the Web, damaging an individual or a hospitals’ online reputation very quickly and with little effort.

**Table 3:** Types and descriptions of different media sharing sites.

Media sharing site category	Example	Description
Video sharing	YouTube	Video sharing site where users can upload, view, share, and comment an unlimited number of videos in both analog and high definition resolutions.
Photo sharing	Flickr	Image and video hosting site with an online community centered on its users and the theme of uploaded photos.
Presentation sharing	SlideShare	Slide sharing site where users can upload presentations in MS PowerPoint, Keynote, Open Office, and .pdf formats.
Document sharing	Scribd	Document sharing site where users can upload different types of document, presentation, and spreadsheet formats.
Music sharing	MySpace	MySpace was the largest social networking site until 2008; however, today MySpace is primarily used as a niche media-sharing site for musicians and emerging artists, which allows them to upload and sell single music tracks and entire albums in MP3 format.
Education sharing	iTunesU	A podcasting service provided through the Apple Corp. iTunes Store which grants free and paid access to educational documents, audio, and video. Content is multidisciplinary and available from kindergarten all the way through university; it includes course lessons, lectures, labs, and lab demonstrations.
Video and images in medicine	Medting	A Web and mobile platform that allows physicians to share medical images and build clinical cases to foster inter and intra institutional collaboration.
Theme specific	The Doctors Channel	Medical video site that offers free CME, medical news, and physician education videos from experts in over 50 specialties.

### Multi-User Virtual Environments and Other Social Media

**Figure 6:**
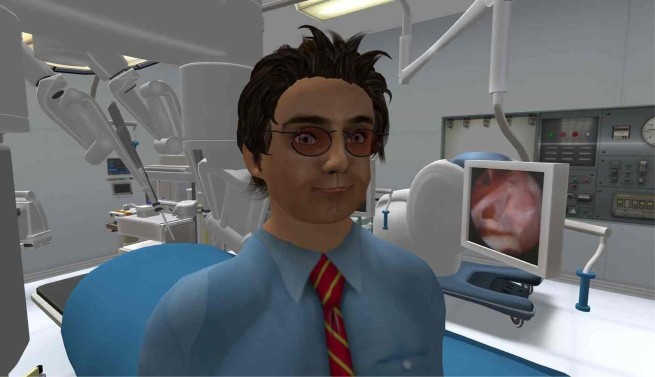
An avatar inside a virtual operating room in Second Life.

Although a large number of social media sites and functionalities are likely to continue emerging, the only remaining category of social media that has not yet been discussed is Massively Multiplayer Online Games (MMOG), more recently branded as Multi-User Virtual Environments (MUVE). These 3-dimensional ecosystems are analogous to a mashup of video games and wikis, which allow users to interact with each other through a virtual representation of themselves known as an avatar. Figure 6 displays an avatar inside a virtual operating room in Second Life.

MUVEs can be classified in two general categories: general purpose and health care specific. The most prevalent general purpose MUVE is Linen Lab’s Second Life, which can be used for gaming or health care education with equal ease. Health care specific MUVEs are less common, typically focusing on particular activities such as medical education (eg, CliniSpace), simulation (eg, OpenSim), and psychiatric treatment (eg, InWorld Solutions). A large body of research exists on the use of MUVEs and is summarized below, particularly focusing on SecondLife.

Historically, MUVEs evolved from early role-playing games. These were text-based and played by multiple users through networked computers; however, computer graphics today allow live rendering that “feels” quite life-like. MUVEs are programmed to simulate many aspects of “real life” in 3 dimensions; thus, when 2 avatars walk closer together, the computer’s user will experience the opposite character’s voice getting increasingly louder, as in real life. This effect is also mimicked graphically; that is, other avatars (and their surroundings) are rendered with increasing sharpness and become more life-like in their interactions as they get closer together.

Some special-purpose MUVEs can even integrate the use of external sensors (eg, built in webcams in laptop computers) to replicate the user’s emotions on their avatars (eg, smiling) [78] and experiments are already underway to incorporate scent, temperature, robotics, and even remote-controlled haptic devices. These extra gadgets have the goal of expanding current MUVE capabilities to add a “fourth dimension” (4D) [79].

Evidence for the use of MUVEs in medicine is growing rapidly with applications in health care [80] and patient education [81], epidemiology [82], mass prophylaxis simulation [83], psychotherapy [84], and research [85].

A paper by Hansen [86] has summarized the major strengths and weaknesses of these environments, which are applicable to both general purpose and health care specific MUVEs. Their strengths lie in their ability to be accessed from the comfort of a user’s own home at any time and their pedagogical flexibility allowing users and content creators with knowledge of the Linden Scripting Language to design and construct a unique environment that mimics “real world” architecture. Their dynamic nature also supports collaboration at a distance, analogously to telemedicine. This is not without cost, however, as the technical barrier to entry in terms of usability often prevents and frustrates most users of these tools (eg, users have a hard time manipulating the avatar on the system and teleporting it to a virtual hospital). Other weaknesses of MUVEs also include the large amount of time required to build a 3D rendering of a physical place and the low efficiency associated with sharing text, images, and videos to an avatar, when compared with standard Web browser-based interfaces of other social media. Finally, the fact that MUVEs are often perceived as computer games, rather than serious clinical and social environments, can also affect their adoption within health care institutions.

Beard and colleagues also conducted research that surveyed health-related activities on Second Life [87]; they found 68 health-related locations. Other notable findings of this paper include the fact that research has demonstrated that using MUVEs can have real-life behavior implications. Indeed, this is the premise behind the US CDC education center on Second Life, which aims to engage visitors to influence real-life health decision-making.

## Discussion

### Ethics, Professionalism, Privacy, and Confidentiality

The potential violation of ethical standards, patient privacy, confidentiality, and professional codes of practice, along with the misrepresentation of information, are the most common contributors to individual and institutional fear against the use of social media in medicine and health care.

Equally important but less well understood is the notion of how these issues vary according to geographical and cultural norms, and how clinicians may protect themselves during Internet-based interactions. A simple example of this is the Tweetspreekuur Dutch primary care consultation service on Twitter, which is considered to be an unethical use of technology by most professional bodies who discourage or prohibit the use of social media for patient-clinician interactions altogether [13,88].

More specifically, there are varied and evolving philosophical views by professional bodies both supporting and condoning the use of social media. These contradictions are further perplexed by regional (eg, health authority) and institutional (eg, hospital) variations in policy. Fundamentally, however, the fear of the unknown appears to be a major barrier against the adoption of social media in clinical settings. This “unknown” is likely due to the conservative nature of health care institutions and practitioners, a lack of understanding of the true risks and liabilities that could result, as well as the question of whose recommendations and best practices should be followed (eg, the Canadian Medical Association [89] supports the conservative use of social media while the British Medical Association [88] and American Medical Association [13] condemn it).

Despite this uncertainty, Hrynaszkiewicz and colleagues [90] recommend that if information is posted publicly, it should not include patient identifiers (eg, patient names, insurance numbers, photos) without written consent. However, if permission is not obtained, clinicians can remain on solid ethical grounds by disclosing up to a maximum of three indirect patient identifiers (eg, sex, disease, treatment).

In general, there is a trend in the literature that recommends all clinicians to ignore patient requests sent through social media [88]. This is important because one could argue that, by not responding to these requests, clinicians are committing an Act of Omission, as there would be implied consent to respond through the medium given that the patient started the dialogue on social media (eg, Twitter).

In terms of social media policies, Boudreaux’s social media governance site [91] is the most comprehensive public database of institutional policies on the subject. As of January 2013, this resource included 219 social media policies, of which 21 were from health care institutions, including stakeholders like the Mayo Clinic, Kaiser Permanente, and Roche. In 2010, research by Kind and colleagues [92] evaluated all US accredited medical schools (n=132) for their social media presence. They found that although 95% (n=126) of American medical schools had a Facebook presence, only 13 had social media policies and only 7 of those encouraged the thoughtful and responsible use of social media.

Research has shown [93,94] that the numbers of privacy and confidentiality violations committed by physicians who use social media are small. For example, Thompson and colleagues at the University of Florida [94] evaluated 1023 student and medical residents’ Facebook profiles in 2007 and 2009. They found that medical students were more likely than residents to violate privacy; however, the only privacy violations that were found were photos of medical mission trips where clinicians were interacting with patients. Even then, out of the more than 1000 profiles evaluated, only 12 ethical violations were found, which accounted for less than 2% of physician profiles. Similar research using content analysis was also conducted with self-identified physicians on Twitter by Chretien [93], who found that out of 314 physicians, each with more than 500 followers, only 3% of their total tweets could be considered unprofessional and 0.7% of them represented potential privacy violations. Nevertheless, even if violations seldom occur, health care professionals should always protect patient privacy and confidentiality as it is the ethos of the medical profession.

### Information Quality

The notion of health-related information quality has been a heated topic of discussion since the mid-1990s when Internet became accessible to the public. Of concern are not only child protection and antipornography initiatives but also quackery and e-pharmacies, which often use social media for direct to consumer (DTC) advertising [95,96]. Equally important are questions of identity theft, misrepresentation of identity (eg, someone falsely claiming to be a medical doctor), and the validity of information that is provided within and through social media.

To establish the validity of the information provided, clinicians who use social media use one or more of the following tactics. First, they take pictures of themselves in a clinical environment and upload them publicly using a service such as Flickr or Picassa, so that any user with access to the Internet can see them. Second, they complete a (public) professional networking site profile, such as LinkedIn, which denotes the location and year of their medical training, professional connections and affiliations, and other credentials or interests. (It is important to remember that professional connections or “friendships” on professional networking sites may give a sense of validation from third parties under false pretenses.) Third, they apply to and are congruent with one or more information quality policy consortiums such as the Healthcare Blogger Code of Ethics (MedBloggerCode) [97] or the Health On the Net (HON) [98] Foundation’s information quality initiative, which allow people to display a digital “ribbon” on their websites with a link to a third-party site that verifies compliance with their principles of information quality.

The principles of information quality, as agreed by HON and MedBloggerCode have been in existence for over a decade; however, they are still questionable because inaccurate and false information is difficult to monitor and police. Even so, most information quality “verification” bodies are reactive rather than proactive when their principles are violated. Verified websites displaying approval ribbons must typically provide the following information: (1) (professional) perspective (eg, is the blogger a cardiologist or a cardiac surgeon?), (2) confidentiality (eg, is patient privacy being protected?), (3) conflicts of interest (eg, is the writer being paid by the pharmaceutical industry?), (4) reliability (eg, are there citations to peer-reviewed material?), (5) courtesy (eg, is third-party content attributed?), (6) purpose (eg, is the purpose of the site clearly stated?), (7) justification of claims (eg, what is the level of evidence behind the information provided?), and (8) contact information (eg, are the contact details of the article author and website publisher accurate?) [97,98].

In contrast, clinicians who use social media anonymously typically use the quality of their content and the minute details provided in their rants to prove the validity of their claims and (to a point) credentials.

At the time of this study, the WHO headquarters in Geneva, Switzerland, is leading a request to the Internet Corporation for Assigned Names and Numbers (ICANN), which manages all domain names on the Internet, for a new specific .health domain. The acquisition of this domain would be strictly legislated and monitored according to quality criteria, such as those noted above, and later prioritized by a consortium of industry partners (eg, Google) to come up as the first search results when people look for health-related information [99]. Theoretically, this would improve consumer confidence with regard to the quality information from the start and improve information trust as a whole because one could validate content from social media sites directly from their Web address. However, whether this initiative will happen is a political issue that requires the support of at least 99 of 198 member states at the World Health Assembly (WHO’s governing body) and will likely not be resolved in the near future.

Validated information sites have also existed since the dawn of the Internet. In social media, wikis like Medpedia (a Harvard, Stanford, University of Michigan, and UC Berkley initiative), which verify authors’ credentials before allowing them to generate content, have tried to improve information quality and “validity”. However, when the majority of articles by these “author verified” sources are compared with open initiatives like Wikipedia, they tend to be shorter, of equal or less quality, and have fewer references due to the restriction of users that can add and democratize the amount of content available on the Internet [100,101].

Videos and multimedia in social media create new challenges but also offer new solutions, such as steering consumers to higher quality information through peer ratings and other forms of “apomediation” [102,103].

### Unanswered Questions

Developing an appropriate “standard of care” involving digital interactions, particularly those over social Web tools are likely to remain a misty ether of agreements due to the range of philosophical, cultural, social, and political values that can be found in the health sector. Professional standards, outlining whether to separate or merge clinical and personal identities are a recurring issue, as health providers have different levels of digital literacy and educational credentials (eg, should nurses have different digital interaction standards from physicians?) [104]. For example, while some professionals may deem it acceptable to use one Facebook profile for both work and personal purposes by using due diligence and monitoring their privacy settings carefully (eg, with whom they share specific information; patients vs close friends), others may lack the technical knowledge necessary to separate their personal and professional life and may have more than one profile or will avoid interactions with current and former patients altogether [95].

There is also a question of whether legal frameworks from telemedicine can or should be adapted to social media. In telemedicine, for example, the common practice is for clinicians to be licensed in the location where the patient is receiving treatment [105]. However, the Internet transcends geographical boundaries, making it nearly impossible to follow the same precedent. Even so, if the patient discloses their address of residence but is physically in a different geographical location when receiving treatment, it is debatable whether this principle should be maintained.

Other issues that need to be addressed, in terms of liability and malpractice, is whether a health care provider, layman, or digital platform should be held liable for health-related recommendations provided through social media [95]. The articulation of policies that are adaptive to the rate of newly evolving social technologies will also continue to be a challenge for decision makers. At the core is the question of whether professional organizations (eg, the British Columbia College of Physicians and Surgeons or its equivalent in other local jurisdictions) would prefer to monitor and enforce every digital interaction or whether they will grant the discretion necessary for their members to exercise their professional judgment and due diligence and undertake an investigation only when they receive a complaint.

Furthermore, there is a need for an urgent evaluation of policies by key actors (eg, Public Health Agency of Canada, the Canadian Medical Association, Provincial Ministries of Health, etc) that are responsible for safeguarding computer-mediated communication in health care. Should, for example, standardization and verification of medical licensing be implemented on the Web and be linked to local licencing bodies? If so, it would need to be operationalized in such a way that provincial colleges of physicians, nurses, and other care providers can link to a database or Web-ribbon to prove their licensed clinician status. Ensuring the highest possible safety and effectiveness of digital interactions is a mutual responsibility of industry, professional associations, and government; however, no hierarchy of responsibility and accountability presently exists. The gaps in policies must be harmonized through a multistakeholder meeting or clinicians will continue to operate in a conflicting policy environment, which may ultimately lead to legal action as a result of their social media use.

Governments also need to identify what business and data-usage models are appropriate in the health sector. For example, is it appropriate to sell patient information? Traditionally, it has been appropriate if multiple patient data are aggregated and anonymized. However, given that users seldom read the terms of service when signing up for a social tool and that they are not allowed to modify them, an ethical question remains about whether they are being de facto coerced to give their data away when joining a specific service. This issue is further complicated by the notion that interacting through social media is an increasing social expectation [106].

An additional issue is that few Web companies and social media service providers are fully transparent, from the moment a user signs up for a social service, about how they will use a user’s data. Even if they are transparent in how they will use the data, the terms of service, which are legal binding documents, often change without the end user having any say in the matter or even being aware of the changes. It is important to consider whether or not users and industry would be willing to open a pay-for-privacy business model, which could potentially allow social media to become an ecosystem for safe and secure digital interactions in health care by allowing clinicians and patients to use services they already use (eg, Facebook) for a safe and ethical health care-related encounter. Alternatively, the question of whether governments should institute a legal requirement for user privacy in these sites will be important to consider in the future, as secure messaging platforms in the health care space are expensive and sometimes even subject to privacy and confidentiality breeches themselves [107].

To date, no longitudinal evaluations of the full economic effects of social media on the health domain have been conducted. Though this may be due to the novel nature of social media, such evaluations would help determine the appropriate incentives (eg, CME vs money), who should provide them, the return on investment, total cost of ownership, scalability, and long-term financial feasibility of using social media [108].

Other theoretical and pragmatic questions must also be addressed, including (but not limited to) the following: (1) Will Wikipedia and other medical wikis that use crowdsourcing and open structures of community-regulated validation become more powerful and sustainable than UpToDate-like resources that have traditionally used a small (paid) group of individuals to create clinical information summaries? (If so, what are the ethical and legal responsibilities of the Wikipedia-like actors towards health consumers?); (2) How biased are social media in providing medical information to users, and is it leading to near-infinite segregation of users around a specific belief (eg, quackery)?; and (3) What are the sociocultural, ecologic, and architectural considerations that must be contemplated over the next decade in the use of social technologies in health care?

### Limitations

This study has a number of limitations. First, results were limited to the English language. Second, during the environmental scans and grey literature queries, snowballing was used, which is subject to friendly and frequent author bias. Third, due to the complexity of the data synthesis process, there was a 3-month lag between the data collection and the completion of this manuscript (despite efforts to monitor new applications and tools during this time, it is possible that new developments during this time period may have been missed, such as [109]). In light of the rapid development in this domain, this time lag from literature collection to manuscript compilation and eventual publication can be significant.

### Professional Implications

#### Overview

This research has demonstrated the many ways that clinicians (as well as patients, health care organizations, and other related stakeholders) can use social media in health care and, as previously noted, that many ethical and legal issues remain unclear. Nevertheless, there are examples of social media demonstrating benefits to patients. Thus, short of having standards and boundaries set by health policy makers and licensing bodies to govern health professional behavior, the following four guidelines may be used to mitigate risk during such interactions over social media and most particularly, as it applies to clinicians.

#### Principle 1: Maintain Professionalism at All Times

Clinicians must remember and follow their institution’s and professional association’s social media guidelines in all digital interactions. If such bodies have not yet created a policy on the use of social media, clinicians must assume that all information exchanged is public and posted in a medium no different than a newspaper. If in doubt about whether the information to be posted is appropriate, it should not be posted. It is also essential to remember that just because a message is private (eg, a direct message on Twitter or Facebook) this does not mean that the information being exchanged is secure and protected. Clinicians and organizations may also use disclaimers to note that the information provided through social media does not indicate any form of endorsement or validation by third parties and that all views expressed are solely those of the author and not those of the institution that the clinician is affiliated with. Indeed, although disclaimers in general have no legal weight in court [78], they do inform the public of separate personal and institutional identities.

#### Principle 2: Be Authentic, Have Fun, and Do Not Be Afraid

The only way to create meaningful relationships over social media is to be genuine. Clinicians should not be afraid to be themselves, so long as they keep in mind Principle 1 and remember the public nature of social media, as well as who their audience is.

#### Principle 3: Ask for Help

People who use social media are very enthusiastic about new members joining their community; thus, clinicians should look for people with similar interests, both professional and personal, and ask for help. Attention to detail should also be placed on how people interact (eg, netiquette) and mimic the social media service and community’s practices (so long as they are professional).

#### Principle 4: Focus, Grab Attention, Engage, and Take Action

One of the most useful models for the successful engagement of an online audience with social media is the Dragonfly Model [110]. By using the analogy of a dragonfly, which needs all four wings to work in concert, equally this model uses the following principles: (1) focus (eg, identify a single, concrete, and measurable goal for using social media), (2) grab attention (eg, make others look at content by saying or posting something interesting), (3) engage (eg, foster personal connections by discussing your interests with like-minded people), and (4) take action (eg, enable and empower others).

### Conclusions

The role of social media in the health care sector is far-reaching, and this article has discussed what, where, how, and why different social media are used in a spectrum of health care–related settings. Questions and debates in terms of governing social media and its applications to medicine and health care are likely to remain contentious, or at least unclear, for some time to come.

Although research has shown that few physicians who use social media violate privacy and confidentiality standards, it is unclear as to whether it is appropriate to delegate discretion to the physicians and allow them to decide if social media is appropriate in specific medical contexts. Indeed, this is the case in the Netherlands with the primary care Twitter consultation service @tweetspreekuur, where Dutch telemedicine policies allow physicians to make the call of whether a particular technology is appropriate for patient care. Understanding which actor or actors are responsible and/or liable, as well as how ethics, confidentiality, privacy, and information quality should be managed will remain central issue that must be resolved in the coming years.

The four guidelines offered here provide a starting point for health care professionals who wish to use social media in a safe and ethical manner. However, much work remains to be done in understanding the pertinence of social media in public care when contrasted with their use in private systems where social media is principally used as a marketing technique to supplement concierge-medicine. Finally, more research will allow us to understand the synergies between social media and evidence-based practice, ultimately allowing for evidence-based policies and economic analyses on the return of investment of using social media.

## Multimedia Appendix 1

Top medgadget blogs during 2010.

## Multimedia Appendix 2

A glossary of commonly used terms on Twitter.

## Abbreviations

API: application programming interface
CDC: Centres for Disease Control
CME: continuing medical education
DNA: deoxyribonucleic acid
DTC: direct to consumer
GDocs: Google documents
HIPAA: Healthcare Insurance Portability and Accountability Act
HIV: human immunodeficiency virus
HLCanada: Health Librarianship Wiki of Canada
HON: Health on the Net
HPV: human papilloma virus
ICANN: International Corporation for Assigned Names and Numbers
MedBloggerCode: Healthcare Blogger Code of Ethics
MMOG: massively multiplayer online games
MUVE: multi-user virtual environment
RSS: really simple syndication
SMS: short message service
WHO: World Health Organization
WHOSIS: World Health Organization Information System
