# Supplementary material for: Social Media: A Review and Tutorial of Applications in Medicine and Health Care
Source: J Med Internet Res. 2014 Feb 11;16(2):e13. doi: 10.2196/jmir.2912 (PMC3936280; doi:10.2196/jmir.2912)
Supplement: Supplementary file 1 [file jmir_v16i2e13_app1.pdf]

Multimedia Appendix 1. Top Medgadget Blogs During 2010.

Blog Category	Site Name	Site Description
Best Medical Blog	EMCrit blog	This blog by an Intensivist from New York City exposes interesting issues in the practice of Emergency Medicine. The site also features podcasts discussing procedures, checklists, and other factoids about the ER.
Best New Medical Blog	ZDoggMD	This humorous blog by a hospitalist in Silicon Valley features music videos, poems about Santa with gout, and other funny things for clinicians in an emotionally tense environment.
Best Literary Medical Blog	StorytellERdoc	This anonymous blog is written by an ER physician who writes about the grace, passion, and sadness of medical practice and the shared humanity between practitioners and patients in the ER.
Best Clinical Blog	GeriPal	This group blog about geriatrics and palliative care features evidence-based reviews. The site also invites (new) qualified authors to contribute their content and has developed into a community with passion and focus.
Best Health Policies/Ethics Blog	Covert Rationing Blog	This blog discusses the various facets of health care rationing; including causes, effects, and how to address underlying factors. The author is a retired professor and researcher in cardiology and cardiac electrophysiology.
Best Medical Technologies / Informatics Weblog	Science Roll	This blog features news and brief reportage-style commentaries on everything from computers to patients and doctors. Other resources are also available on medical informatics and genetics.
Best Patient's Blog	Wheelchair Kamikaze	This patient blog covers a spectrum of issues from living with Multiple Sclerosis to research news and lifestyle articles. The author is regarded as an authority for patients with MS and fosters a support community with his blog posts.
