# Supplementary material for: Social Media: A Review and Tutorial of Applications in Medicine and Health Care
Source: J Med Internet Res. 2014 Feb 11;16(2):e13. doi: 10.2196/jmir.2912 (PMC3936280; doi:10.2196/jmir.2912)
Supplement: Supplementary file 2 [file jmir_v16i2e13_app2.pdf]

Multimedia Appendix 2. A Glossary of Commonly Used Terms on Twitter.

Terminology	Definition
@	The @ sign is used to call out other users within a tweet. When a username is preceded by the @ sign, it becomes a link to a Twitter profile.
Avatar	The personal image uploaded to a Twitter profile.
Block	To block someone on Twitter means they will be unable to follow you or add you to their lists, and will not deliver their mentions (see below) to your mentions tab
Direct Message (DM)	These are private Tweets between two users.
Favorite	A tweet marked as favorite saves the message for later viewing.
Follow Count	The number of people that follow a user, as well as how many people that user follows.
Follower	A follower is a separate Twitter who follows "you".
Follow Friday	The most commonly used hashtag #FF, it recommends who other users should follow based on personal experience and interactions with other users. #FF is used on a Friday and it is a form of social courtesy.
Following (verb)	The act of following another user.
Geotagging	Tagging (metadata) added to a tweet, which denotes the geographical location from where the message was sent; enabling this feature allows users to disclose where they are in real-time.
Handle	A user's "Twitter handle" is the username they have selected and the respective accompanying URL (e.g., http://twitter.com/username
Hashtag	The # symbol is used to mark keywords or topics in a Tweet. It serves to search the twitter sphere on a particular topic.
Listed	To be included in another Twitter user's list.
Lists	These are publicly or individually curated groups of Twitter users. They are often used as a filter to find tweets of users who follow a particular topic (e.g., Cardiologists)
Mentions	Mentioning another user in a tweet by including their handle.
Reply	A Tweet posted in reply to another user's message, usually posted by clicking the "reply" button next to their Tweet within a timeline. If the users' twitter timeline are public, it allows people to follow the tweet conversation and response thread.
Retweet (noun)	A Tweet by another user, forwarded to you by someone you follow. Often used to spread news or share valuable information and allows it to spread virally.

Terminology	Definition
Retweet (verb)	The act of forwarding another user's Tweet to a user’s personal followers
Search	A function of twitter (found at http://search.twitter.com) to search for usernames, hashtags, or a particular subject. (This function is generally limited to the past 2 weeks of public tweets.)
Short URL (SURL)	URL shorteners are used to turn long web addresses (URLs) into shorter URLs (e.g., http://ow.ly/exampleshortlink).
Spam	Unwanted messaging or following on Twitter.
Timeline	A real-time list of Tweets on Twitter.
Tweet (noun)	A message posted via Twitter containing 140 characters (or fewer).
Tweeterer	An account holder on Twitter who posts and reads Tweets.
Unfollow	To cease following another Twitter user. This will cause their Tweets to no longer show up in a user’s home timeline.
Widget	A discrete code that allows a twitter user to feature their “tweets” in real time on a different website (e.g., a blog).
